# Supplementary material for: Functionalized Ordered Mesoporous MCM-48 Silica: Synthesis, Characterization and Adsorbent for CO2 Capture
Source: Int J Mol Sci. 2023 Jun 19;24(12):10345. doi: 10.3390/ijms241210345 (PMC10299513; doi:10.3390/ijms241210345)
Supplement: Supplementary file 1 [file ijms-24-10345-s001.zip › ijms-2425401-supplementary.pdf]

Supporting Information

Functionalized ordered mesoporous MCM 48 silica: synthesis, characterization and adsorbent for CO_2 capture

Silvana Borcănescu, Alexandru Popa*, Orsina Verdeș and Mariana Suba

[1] "Coriolan Drăgulescu" Institute of Chemistry, Bl. Mihai Viteazul No. 24, 300223 Timisoara, Romania
* Correspondence: alpopa_tim2003@yahoo.com; Tel.: +40-256-491818

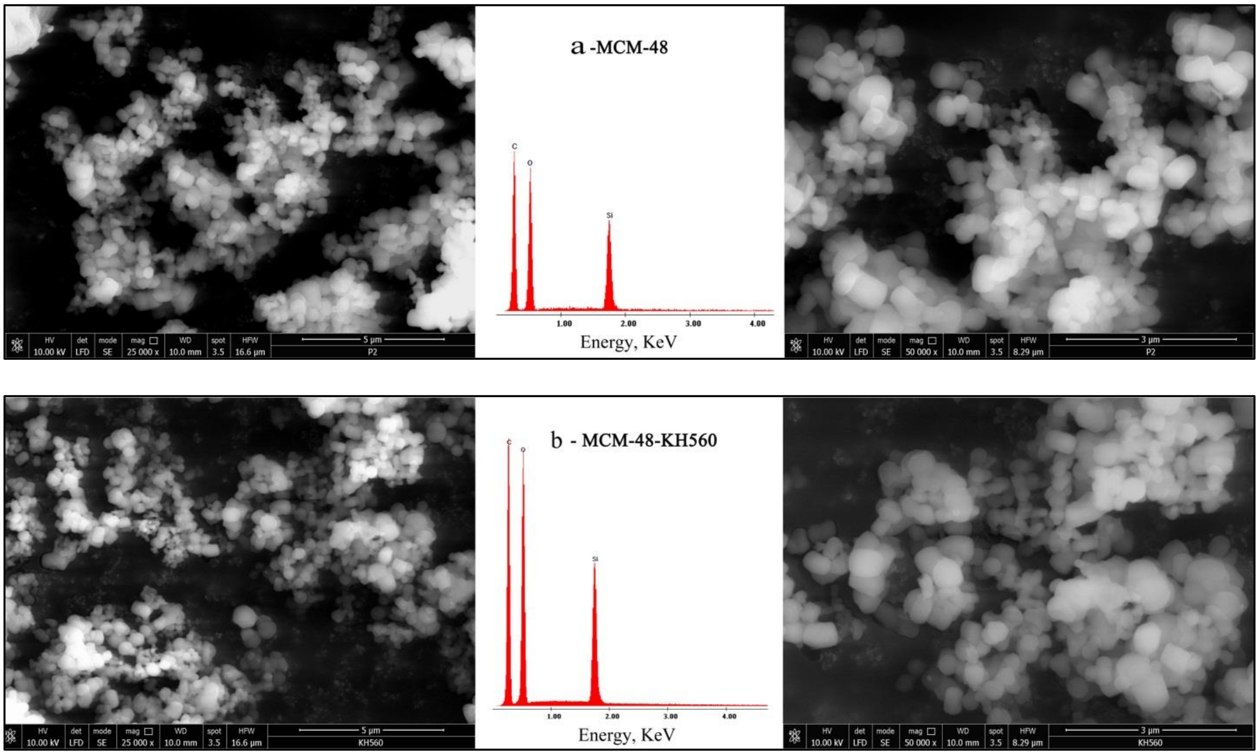

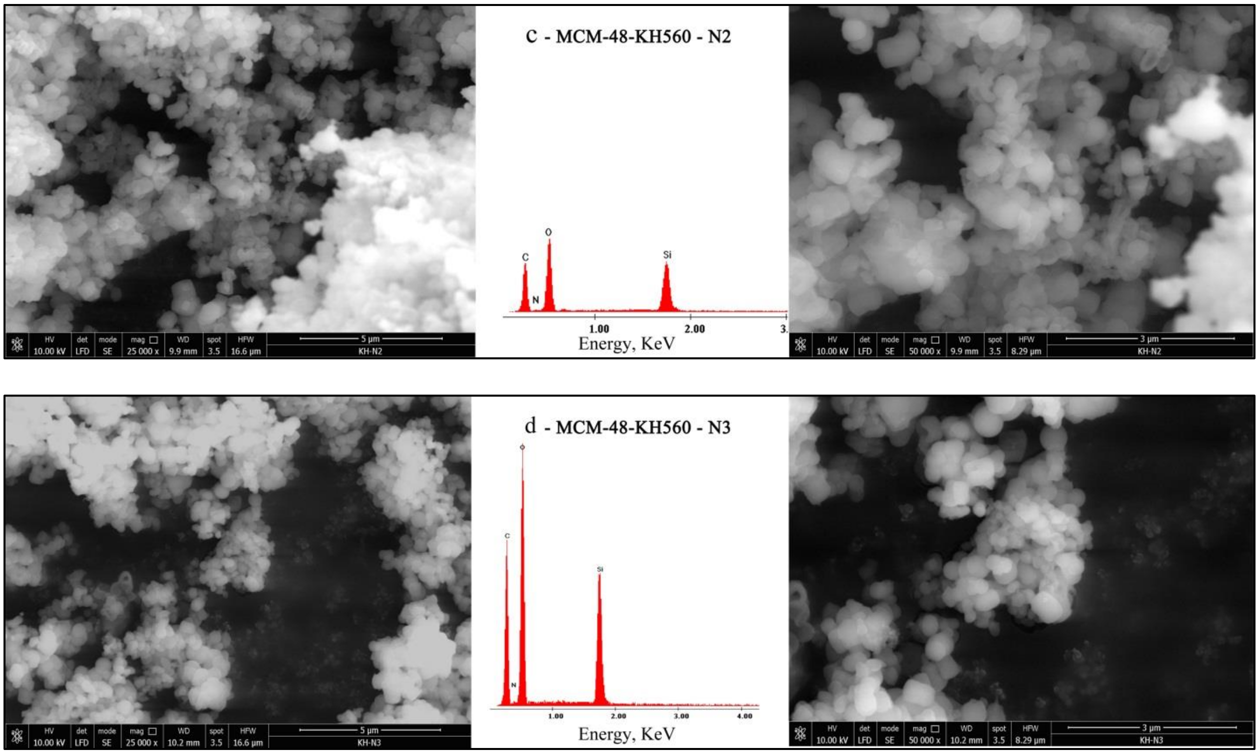

Figure S1. SEM micrographs images of MCM-48 a), MCM-48 sil KH b), MCM-48 sil KH N2 c) and MCM-48 sil KH N3 d) mesoporous materials.

Scanning electron micrographs images were used to analyze the morphology of prepared mesoporous molecular sieves. In Figure S1 a-d, the morphology of the materials is studied by SEM images using different magnifications, more specifically 25000x and 50000x. The materials indicate that micrometric clusters are formed. It can be seen that by increasing the magnification from 25000x up to 50000x, the shape of the particles becomes more clear as spherical. The size of the spheres is around 200-500 nm.

It can be seen that the spherical morphology is still maintained after the introduction of KH560 and after N2 and N3 amine functionalization. After the functionalization of the materials, a change in the EDS spectra can be observed. So, from the EDS images presented in Figure S1 a-d the existence of amino groups in the materials is confirmed by the presence of nitrogen peaks.
